# The Prognostic and Therapeutic Roles of ARL-6 Gene in Hepatocellular Carcinoma

**DOI:** 10.7150/ijms.88039

**Published:** 2024-01-01

**Authors:** Jin Wang, Fuheng Che, Yuanyuan Zhao, Lai Wei, Dong Chen, Chen Dai, Bo Zhang, Xi Zhou, Bo Yang, Zhishui Chen

**Affiliations:** 1 Institute of Organ Transplantation, Tongji Hospital, Tongji Medical College, Huazhong University of Science and Technology, Wuhan, China.; 2 Key Laboratory of Organ Transplantation, Ministry of Education, Wuhan, China.; 3 NHC Key Laboratory of Organ Transplantation, Wuhan, China.; 4 Key Laboratory of Organ Transplantation, Chinese Academy of Medical Sciences, Wuhan, China.

**Keywords:** hepatocellular carcinoma, *ARL-6*, prognostic biomarker, tumor-infiltrating immune cells

## Abstract

**Background:** Hepatocellular carcinoma (HCC) is one of the most prevalent human cancers. *ARL-6*, a member of the ADP ribosylation factor (like) (*ARF*) protein family, has gained attention as a potential therapeutic target in various malignancies and a prognostic biomarker. However, its specific roles in HCC, both prognostically and biochemically, remain largely unclear.

**Methods:** To examine the functional relevance of *ARL-6* in HCC, we acquired data from GEPIA, UALCAN, TIMER, TCGA, GeneMANIA, and Metascape databases. Then, we conducted immunohistochemistry on a replication sample comprising 26 HCC specimens to assess the efficacy of the *ARL-6* gene. To unravel the mechanistic intricacies, we employed diverse assays such as the cell counting kit 8 (CCK8), flow cytometry, and transwell invasion assessment.

**Results:** Our findings demonstrated the mRNA expression of *ARL-6* was significantly upregulated in HCC compared to normal tissue, as evidenced by comprehensive database analysis. Immunohistochemistry further revealed that *ARL-6* expression was remarkably higher in HCC than in para-carcinoma tissues. Moreover, *ARL-6* expression exhibited noteworthy variations across diverse LIHC characteristics, including sample type, histological subtype, *TP53* mutation status, nodal metastatic status, and cancer stage. In addition, high transcriptional levels of *ARL-6* were correlated with diminished overall survival (OS) and disease-free survival (DFS) in HCC patients. Furthermore, our study indicated positive correlations between *ARL-6* expression levels and the activities of tumor-infiltrating immune cells such as B cells, myeloid dendritic cells, macrophages, neutrophils, CD8+T cells, and CD4+T cells. Substantiating our findings, database analysis uncovered additional evidence of *ARL-6* gene co-expression and its functional significance in HCC cases. Finally, we demonstrated the involvement of the *ARL-6* gene in HCC cell invasion, proliferation, and apoptosis.

**Conclusions:** In conclusion, our investigation sheds light on the pivotal role of *ARL-6* in influencing HCC prognosis and treatment by modulating the biological activities of tumor cells. These discoveries hold promise for the development of predictive biomarkers and novel therapeutic avenues for affected patients.

## Introduction

Cancer incidence and mortality statistics reveal the staggering global impact of primary liver cancer, ranking sixth and second, respectively 1. Among these cases, hepatocellular carcinoma (HCC) takes the forefront, constituting nearly 75 % of all liver cancer occurrences and standing as the most prevalent histologic subtype 2. Although HCC ranks as the sixth most fatal cancer among men in developed nations, its gravity reaches a disconcerting level in economically stable regions like China, where it claims the second-highest spot in cancer-related male fatalities 3. Despite the great efforts of the medical community, HCC remains a formidable challenge, primarily due to the complex and enigmatic molecular mechanisms governing its development and progression 4. Consequently, unraveling the pathophysiological intricacies contributing to HCC carcinogenesis becomes an imperative pursuit for the discovery of novel prognostic biomarkers.

The current approaches to HCC treatment are marred by a multitude of issues. Conventional therapies, such as surgical resection, chemotherapy, and radiation, often exhibit limited efficacy, particularly in advanced HCC stages. Moreover, the risk of recurrence appears large, posing a perpetual threat to patients' well-being. The dearth of early diagnostic tools may exacerbate the problem, with HCC often diagnosed at an advanced and less treatable stage, further compromising patient outcomes 5. Prognosis in HCC remains a tremendous challenge, with patients facing uncertain futures, largely dictated by the intricacies of individual tumor biology. This uncertainty exacts a considerable emotional toll on patients and their families, emphasizing the urgent need for reliable prognostic markers that can provide a more complete picture of HCC progression 6.

In the quest to address these pressing issues, the GTP-binding protein *ARL-6* (ADP ribosylation factor-like GTPase 6) emerges as a promising candidate. A member of the *ARF*-like (ADP ribosylation factor-like) family, *ARL-6* plays a pivotal role in intracellular traffic regulation 7. This family's cornerstone, ADP-ribosylation factors (*ARF*s), encompass 20-kDa guanine nucleotide-binding proteins, intricately involved in both exocytic and endocytic vesicular transport processes, even enhancing the ADP-ribosyl transferase activity of cholera toxin. The *ARF*-like family of proteins, including *ARL-6*, exhibits diverse roles in various cancer types. Some members, such as ARL2 and ARL4, are associated with breast cancer, colorectal cancer, endometrial cancer and other malignancies, impacting processes such as cell proliferation and migration, while others like *ARL6IP5* and *ARL6IP1* have relevance in colorectal and pancreatic cancer, respectively 8-10. Although *ARF* family members have been implicated in tumor development through their influence on cancer cell proliferation, migration, and invasion 8, 11-15, the role of *ARL-6* in HCC remains largely unclear. Notably, its expression may hold the potential to serve as a significant biological marker for HCC prognosis.

Thus, our study delves into the expression and functions of *ARL-6* in the context of HCC, aiming to elucidate its potential to impact prognosis by regulating the biological activity of HCC tumor cells. The findings from this investigation not only hold the promise of identifying novel prognostic biomarkers but also herald a new era in the development of targeted therapies for HCC, addressing the urgent need for more effective treatment options in light of the complex molecular intricacies that underlie this formidable disease. Nevertheless, the expression of *ARL-6* and its role within the *ARF*-like family in other types of cancer warrants further exploration.

## Material and methods

### GEPIA Data Analysis

GEPIA (gepia.cancer-pku.cn) is a user-friendly platform that provides access to data from the Cancer Gene Atlas (TCGA) and Genotype-Tissue Expression (GTEx) projects. It comprehensively covers 8,587 normal tissue samples and 9,736 cancer tissue samples. In this study, GEPIA was used to analyze overall survival (OS) and disease-free survival (DFS) outcomes for HCC patients. The survival outcomes between HCC patients with low and high expression levels of specific genes were then compared. This analysis involved identifying patients at risk, calculating hazard ratios (HRs), determining 95 % confidence intervals (CIs), and assessing P-values to elucidate significant associations.

### UALCAN Data Analysis

Our research also involved the use of UALCAN (ualcan.path.uab.edu/analysis), which compiles data from TCGA and the MET500 sequencing project. Specifically, the expression of *ARL-6* in normal tissues, cancer tissues, and different types of malignant tumors were determined based on their prevalence. Student's t-tests were employed to compare statistically significant differences between these groups, with a significance threshold set at P < 0.05.

### TIMER Database Analysis

The TIMER database (cistrome.shinyapps.io/timer) was utilized to systematically investigate cellular immune infiltrations and clinical medical risks. Our analysis focused on the expression of *ARL-6* in advanced liver cancer, as well as tumor purity and immune cell infiltrations (such as dendritic cells, monocytes, macrophages, CD8+ T cells, and CD4+ T cells) within the tumor microenvironment.

### TCGA Database Analysis

The TCGA dataset (portal.gdc.com) was accessed to obtain RNA-sequencing expression (level 3) profiles, TRNA-sequencing expression (level 3) profiles, and corresponding clinical data for HCC. The ggstatsplot tool in R was used to visualize the associations between *ARL-6* gene expression and immunological scores. Subsequently, the relationship between non-normally distributed quantitative data was examined via Spearman's correlation analysis. To predict *ARL-6* mRNA accuracy, time receiver operating characteristic (ROC) analysis was performed. Kaplan-Meier curves were constructed using log-rank tests and univariate Cox proportional hazards regression to calculate p-values, HRs, and 95 % CIs. R version 4.0.3 was employed for all analytical procedures, facilitated by relevant R packages.

### GeneMINIA Data Assessment

GeneMANIA (www.genemania.org) is an online predictive analytic tool that explores protein and genetic interactions, domain protein similarities, co-expression, co-localization, and functional associations within the context of target genes. In this study, we assessed the connections between *ARL-6* and the mutual impact of hereditary genes.

### Metascape Analysis

To explore the functional roles of *ARL-6* and its co-expressed genes, Metascape (metascape.org), a mobile app for genomic annotation and pathway analysis, was utilized. The specific cutoff criteria, including a P-value threshold of 0.01, an enrichment index above 1.5, and a minimum gene count of three, were applied to identify biologically significant associations.

### Clinical Samples

Twenty-six biopsy specimens were randomly selected from Huazhong University of Science and Technology and the Institute of Organ Transplantation at Tongji Hospital, Tongji Medical College. All samples were histologically diagnosed as hepatocellular carcinoma. Ethical approval (TJ-IRB20210938) was obtained from the respective institutions, and informed consent was obtained from all participants.

### Immunohistochemical Staining

Tissue slices from hepatocellular carcinoma were fixed in formalin (Thermo Scientific, Waltham, MA), paraffin embedded (HAS biotech, China), and sectioned at 4 µm thickness. The slices were immunostained with anti-*ARL-6* antibody (Abclonal, China) using a Leica Bond-Max Polymer Refine Detection Kit (Leica Biosystems, Buffalo Grove, IL). Aipathwell (www.servicebio.cn) was utilized to examine the sections. The immunoreactive score (IRS) was calculated as follows: IRS = PP (positive cell ratio) × SI (positive intensity). SI was categorized into three grades: 0, 1, 2, and 3 referring to no, weak (light yellow), moderate (brown), or strong (brown) positive staining. PP was classified into four grades: 0 with 0-5 %, 1 with 6 %-25 %, 2 with 26 %-50 %, 3 with 51 %-75 %, and 4 with & GT.

### Cell Culture, RNAi, and Transfection

The L02, HepG2, Hep3B, Huh7, Alex, SMMC7721, and 97L cell lines were obtained from ATCC (American Type Culture Collection, Rockville, MD, USA) and cultured in high-glucose (4.5 g/l) Dulbecco's modified Eagle medium supplemented with 10 % fetal bovine serum (FBS) at 37 °C in a humidified incubator with 5 % CO_2_. Guangzhou RioboBio (Guangzhou, Guangdong, China) was used to develop and produce the *ARL6* siRNA (*5'-GAATGGTTGTGGCCAAAGA-3'*) and siRNC (*5'-TTCTCCGAACGTGTCACG-3'*). For transfection, the cells (1×10^5^ cells/well) were seeded for 24 h, and then exposed to siRNA fragments and control siRNAs.

### Western Blotting

The cells were lysed in RIPA buffer for 15 min at 4 °C after rinsing with PBS. Subsequently, the lysates underwent centrifugation at 14,000 rpm for 15 min to eliminate debris, allowing for the collection of the supernatants. The concentration of mouse ion proteins within the soluble fraction was determined using the BCA protein assay from Thermo Scientific. The protein samples were separated through SDS-PAGE, and then transferred onto a nitrocellulose membrane. After blocking with a 5 % non-fat dried milk solution in TBST overnight, the target proteins were identified using SuperSignal West Pico Chemiluminescent (Thermo Scientific). *ARL-6* (Abcolonal) was used at a dilution of 1:1000. β-actin (Sigma) served as a loading control at a dilution of 1:3000.

### Cell Viability Assay

The CCK-8 kit (Dojindo) was used to determine cell viability. Huh7 cells were subjected to siRNA transfection following the aforementioned steps. After incubation for 24 h, the cells were plated at a density of 5000 cells per well in 96-well plates. After adding CCK8 solution (10 μL) to each well, the plates were incubated at 37 °C for 100 min. The samples' absorbance values were then determined using a microplate reader at a wavelength of 450 nm. Each experiment was conducted with at least three replicates.

### Flow Cytometry Analysis for Cell Apoptosis

After siRNA transfection, the cells were collected and stained using an Annexin V-FITC and propidium iodide (PI) staining kit (Multi Sciences, Hangzhou, China) to determine the extent to which they had undergone apoptosis. The exact proportions of apoptotic cells were determined by flow cytometry analysis using a FACS Calibur equipment (BD Biosciences). Data analysis was performed via the FlowJo program. Each experiment was conducted with at least three replicates.

### Transwell Invasion Assay

Transwell invasion assays were conducted using BD BioCoat Matrigel Invasion Chambers (Becton Dickinson Labware, USA), in conjunction with Polyethylene terephthalate-based migration chambers. Then, 200 μL of serum-free media was employed to seed the cells onto the Matrigel-coated transwell inserts. Meanwhile, 500 μL of medium with 10 % FBS was placed in the bottom chamber. Following a 24-hour incubation period, the cells that had adhered to the upper surface of the transwell inserts were gently removed using a cotton swab. After a 10-min interval, the invasive cells were stained with crystal violet. Upon staining, the cells were imaged and quantified. Each experiment was conducted with at least three replicates.

### Statistical Analysis

Statistical analysis was performed to assess the significance and reliability of the experimental results obtained from the described assays. Data were expressed as mean ± standard deviation (SD). To evaluate the significant differences between control and experimental groups, a two-tailed t-test or analysis of variance (ANOVA) was employed, followed by post hoc tests if necessary. All statistical analyses were conducted using SPSS software packages, and p-value < 0.05 was considered statistically significant.

## Results

### Abnormal Expression of *ARL-6* Gene in HCC Patients

**Figure 1:**
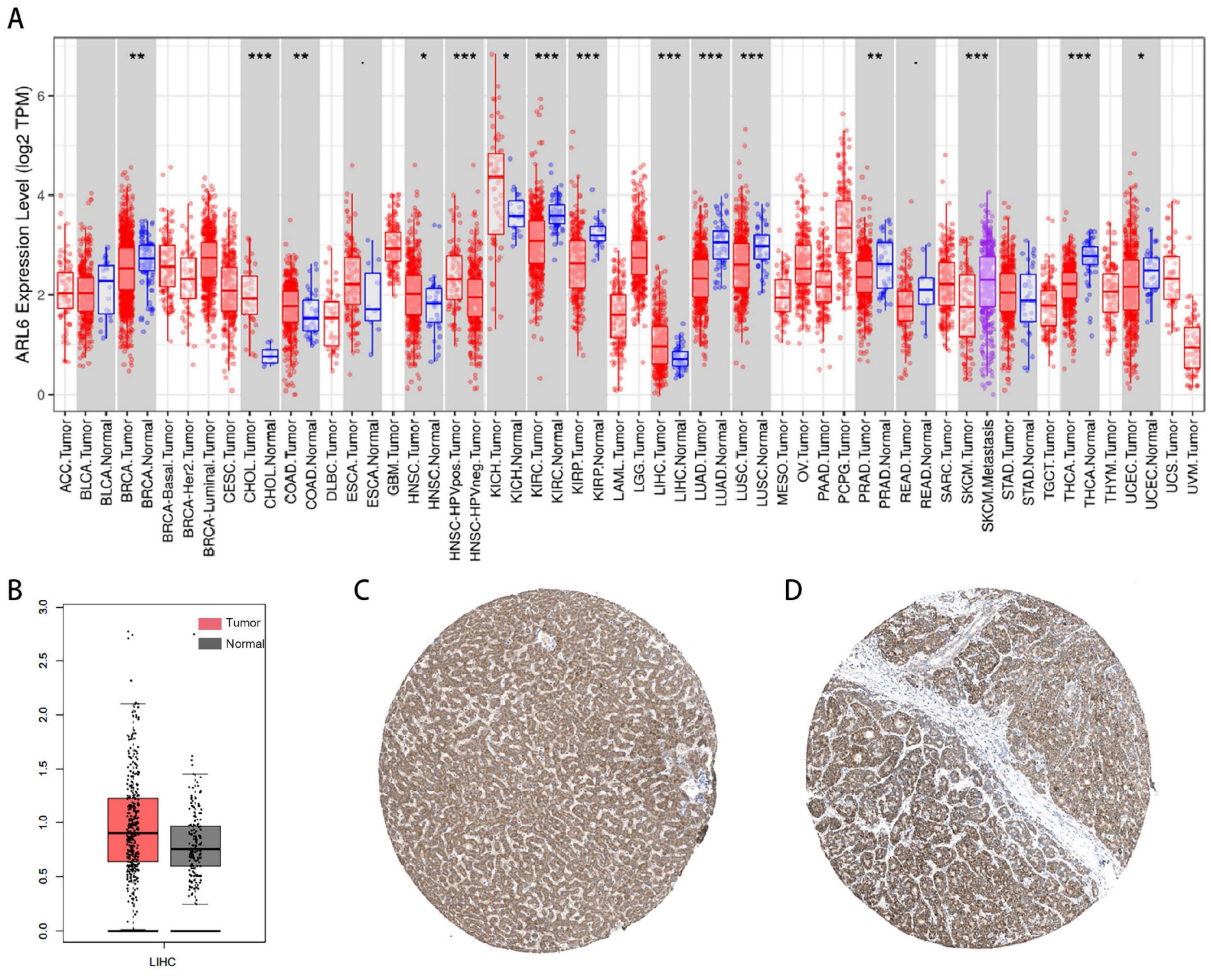
Abnormal Expression of *ARL-6* Genes in HCC Patients. (A) TIMER2 was utilized for visualizing *ARL-6* expression in several tumor types. (B) The GEPIA-determined *ARL-6* gene expression level in HCC is shown as a boxplot. Tumor samples are shown in red; normal samples are shown in gray. Tumor (T) and normal (N) tissue. (C-D) *ARL-6* immunohistochemical findings from the HPA database, showing both HCC and normal liver tissue. Significance: *p < 0.05; **p < 0.01; ***p < 0.001.

The investigation into *ARL-6* gene expression in tumor tissues was conducted using the TIMER database. According to the findings, the mRNA expression of *ARL-6* was significantly higher in various cancers compared to adjacent normal tissues, including stomach adenocarcinoma (STAD), liver hepatocellular carcinoma (LIHC), kidney chromophobe (KICH), head and neck cancer (HNSC), esophageal carcinoma (ESCA), colon adenocarcinoma (COAD), and cholangiocarcinoma (CHOL). In contrast, the mRNA expression of *ARL-6* was markedly lower in UCEC (uterine corpus endometrial carcinoma), THCA (thyroid carcinoma), SKCM (Skin Cutaneous Melanoma), READ (Rectum adenocarcinoma), PRAD (prostate adenocarcinoma), LUSC (lung squamous cell carcinoma), LUAD (lung adenocarcinoma), KIRP (kidney renal papillary carcinoma), KIRC (kidney renal clear cell carcinoma), BRCA (breast invasive carcinoma), and BLCA (bladder urothelial carcinoma) (Figure 1A). Furthermore, according to GEPIA data, the expression levels of *ARL-6* were significantly increased in liver cancer tissue compared to healthy liver tissue (Figure 1B).

**Figure 2:**
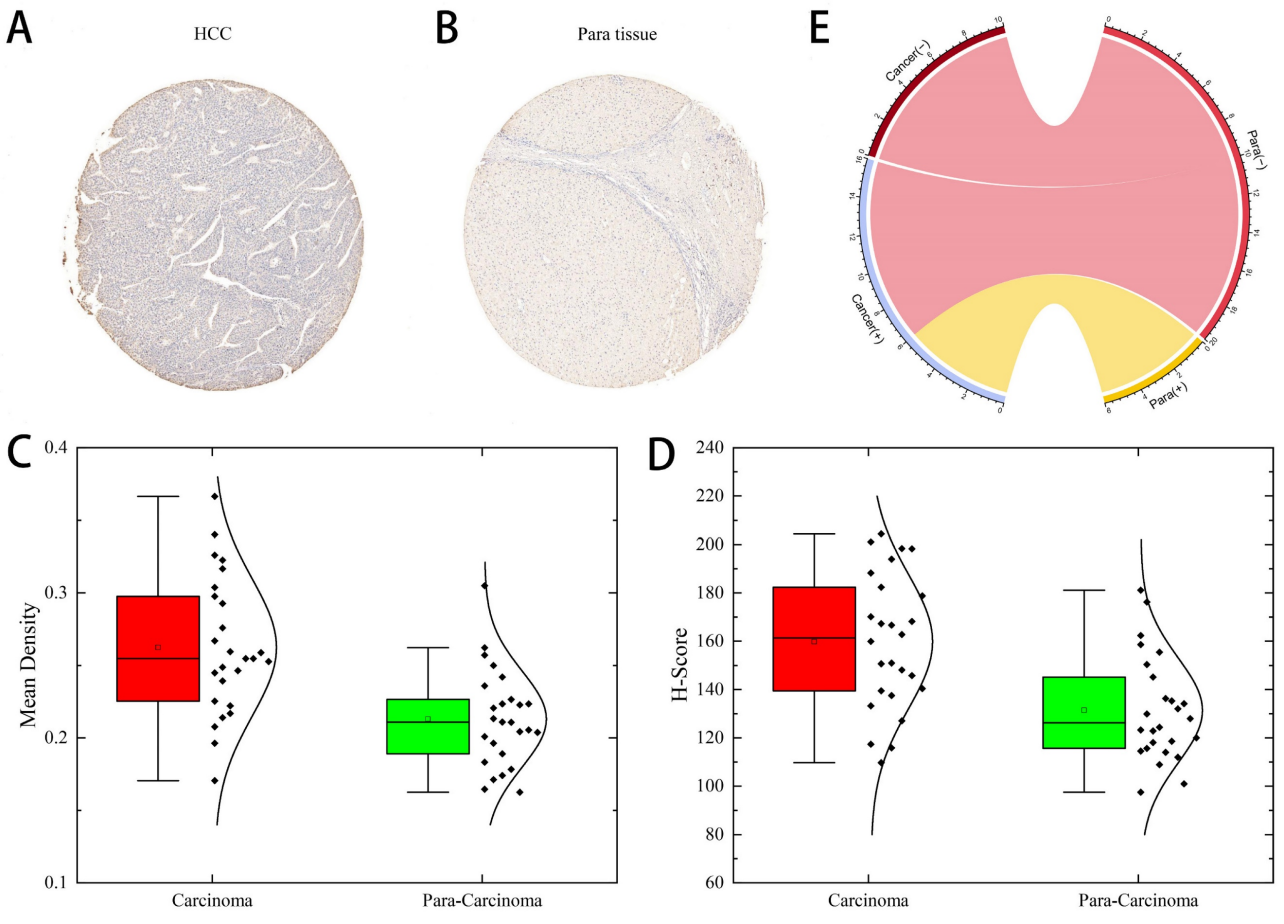
*ARL-6* expression was analyzed by immunohistochemistry in HCC cancer tissue. (A-B) Immunohistochemical staining assay for HCC and hepatocellular para carcinoma tissue samples. (C-E) Comparison of carcinoma and para carcinoma tissues in terms of Means Density, H-score, and IRS.

Next, we determined the protein levels of *ARL-6* in HCC cases using the human protein atlas (HPA) database. Notably, the protein expression of *ARL-6* was higher in HCC tissues compared to normal tissues (Figure 1C). To validate these database findings, the study included 26 HCC tissues and 26 para-carcinoma tissues (Figure 2A-B). A comparison between carcinoma and para-carcinoma tissues was performed (Table 1). Statistically significant differences in Mean destiny (0.26±0.05, t=6.366, P<0.0001), H-Score (159.86±27.99, t=4.759, P<0.0001), and IRS (6.4±1.98, t=3.953, P<0.0001) values were observed. Scatter plots depicting 26 para-carcinoma tissues and 26 cancer tissues are summarized in Figure 2C-E. Taken together, these results indicate that both the mRNA and protein levels of *ARL-6* are overexpressed in HCC cases.

### The Predictive Values of *ARL-6* in HCC Patients

**Figure 3:**
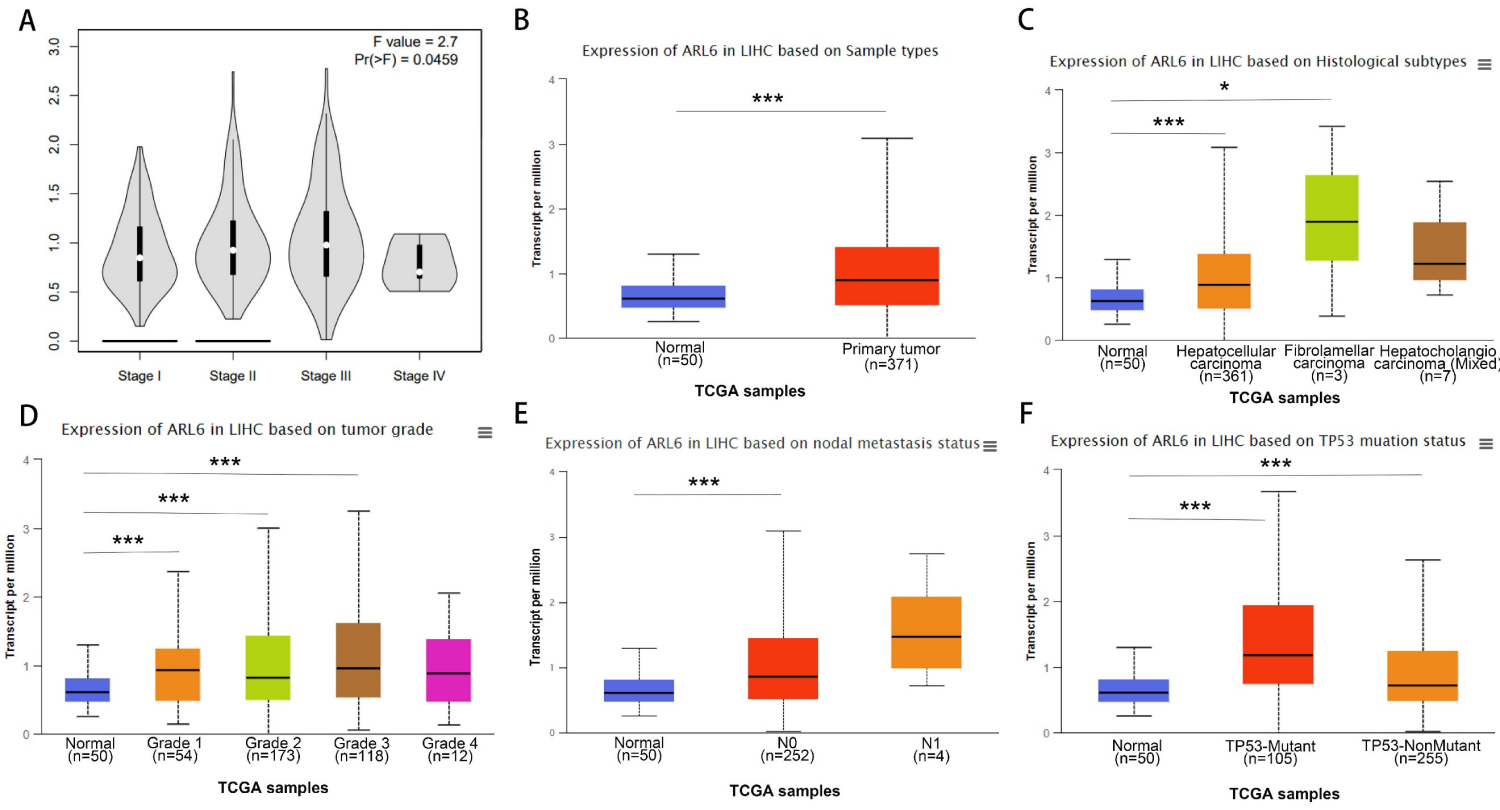
A subset of individuals with liver cancer had their *ARL-6* transcription differentiated by histological subtype, tumor grade, and other factors. (A) Expression of *ARL-6* is correlated with LIHC pathology stages in GEPIA datasets. A series of box-plots showing *ARL-6* expression in (B) liver cancer and normal samples, (C) normal cases with differential histological subtypes of liver cancer, (D) normal cases with stage 1, 2, 3, or 4 liver cancers, (E) normal cases with metastatic tumors, and (F) normal and liver cancer samples based on TP53 mutation status. Significance as above.

Using the GEPIA database, we assessed the relationship between differentially expressed *ARL-6* and the tumor grade of HCC cases to determine the association of *ARL-6* with HCC prognosis, progression, and carcinogenesis. A significant correlation (P=0.0459) was observed between *ARL-6* expression and tumor grade, with the highest expression of *ARL-6* found in grade III tumors (Figure 3A). Additionally, we examined *ARL-6* expression in hepatocellular carcinoma of the liver with various clinical features using the UALCAN database. TP53 mutation status, nodal metastatic status, cancer stage, histological subtype, and sample type were all shown to substantially affect *ARL-6* expression (Figures 3B-F). These results suggest that *ARL-6* is critically involved in HCC carcinogenesis and development.

**Figure 4:**
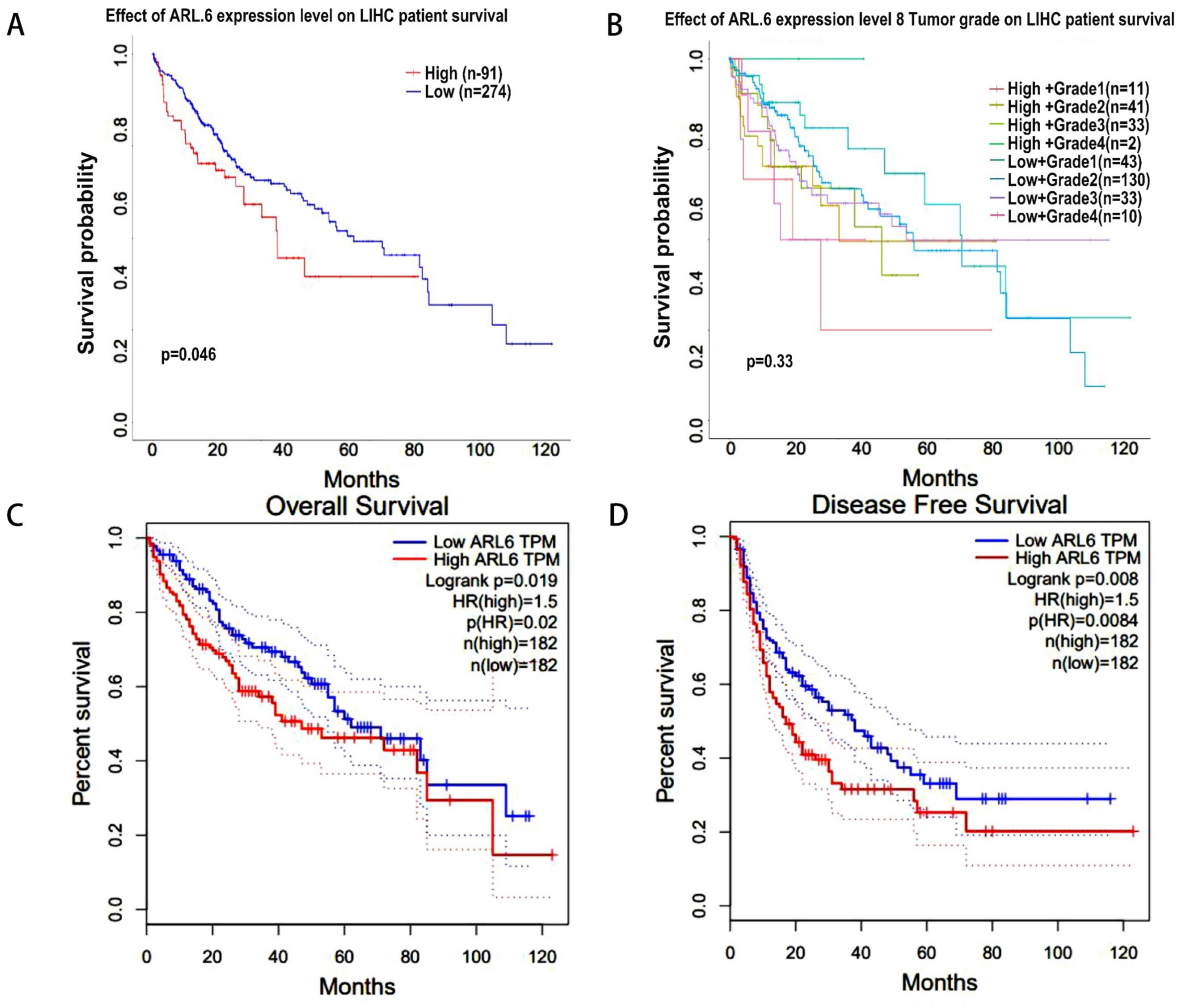
HCC-related survival analysis of *ARL-6* gene. (A) Effect of *ARL-6* expression level on LIHC patient survival. (B) The influence of *ARL-6* expression and tumor grade on LIHC survival. In HCC, *ARL-6* overexpression was correlated with reduced (C) OS and (D) DFS.

Next, using the UALCAN database, we analyzed the correlation between *ARL-6* expression and HCC outcomes. Increased *ARL-6* expression was associated with worse survival in LIHC (Figure 4A). However, the differential expression level of *ARL-6* combined with the tumor grade of HCC patients did not have a statistically significant effect on HCC prognosis (Figure 4B). The OS curve of *ARL-6* in the GEPIA database showed that a high transcriptional level of *ARL-6* was significantly linked with shorter OS in HCC patients (P=0.019; Figure 4C). We also investigated the potential predictive role of *ARL-6* differential expression in the DFS of HCC. High levels of *ARL-6* transcription were significantly correlated with decreased DFS in patients with HCC (P=0.008; Figure 4D).

**Figure 5:**
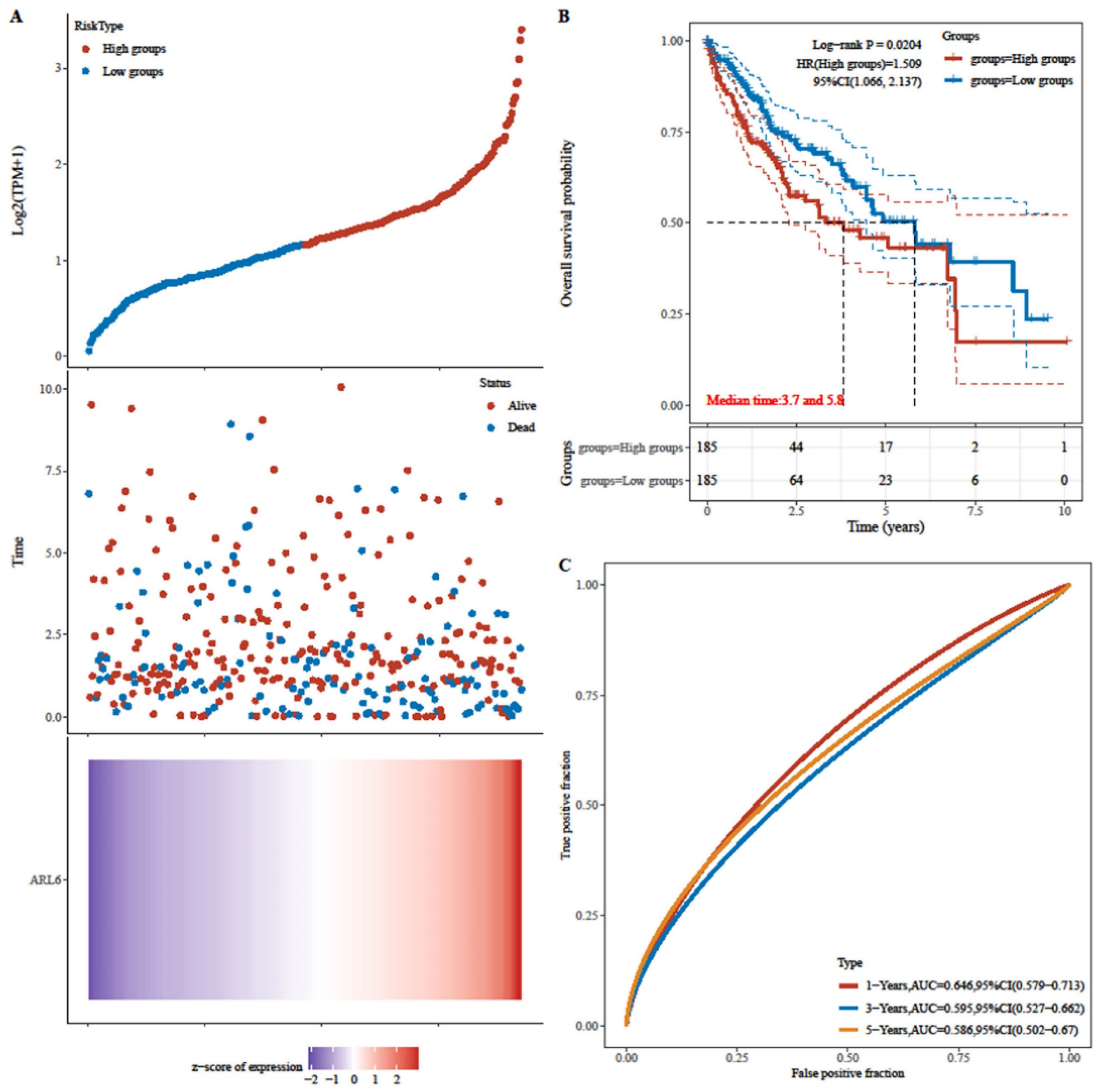
Analyzing the risk scoring model constructed from the differential expression of the *ARL-6* gene in TCGA-HCC cases with respect to its predictive performance and survival analyses. (A) Gene expression heat map for the *ARL-6* gene in the TCGA-HCC cohort with the risk scores and distributions of patient survival times. (B) The TCGA-HCC cohort's OS as compared across risk categories using Kaplan-Meier analysis. (C) The TCGA-HCC cohort's 1-year, 3-year, and 5-year ROC curves for the risk score model used for predicting OS.

Next, patients' risk scores in the TCGA-HCC dataset were determined through *ARL-6* gene expression and regression coefficients. Figure 5A displays the frequency of each risk score in the TCGA-HCC dataset. Cases in the TCGA-HCC group were classified as either high-or low-risk based on their median risk score. Furthermore, the survival time distribution (Figure 5A) indicated that the prognosis of HCC deteriorated along with the risk score. Expression levels of the *ARL-6* gene are also shown in Figure 5A. The prognosis of the high-risk group was considerably poorer than that of the low-risk group, as demonstrated by the log-rank test and Kaplan-Meier analysis (Figure 5B; P 0.05). Figure 5C depicts ROC curves for the effectiveness of the risk scoring system in predicting patient outcomes at one, three, and five years in the TCGA-HCC group. The areas under the ROC curves (AUCs) were 0.646, 0.595, and 0.586 at one, three, and five years, respectively.

### *ARL6* Gene Infiltration by Immune Cells in HCC Cases

**Figure 6:**
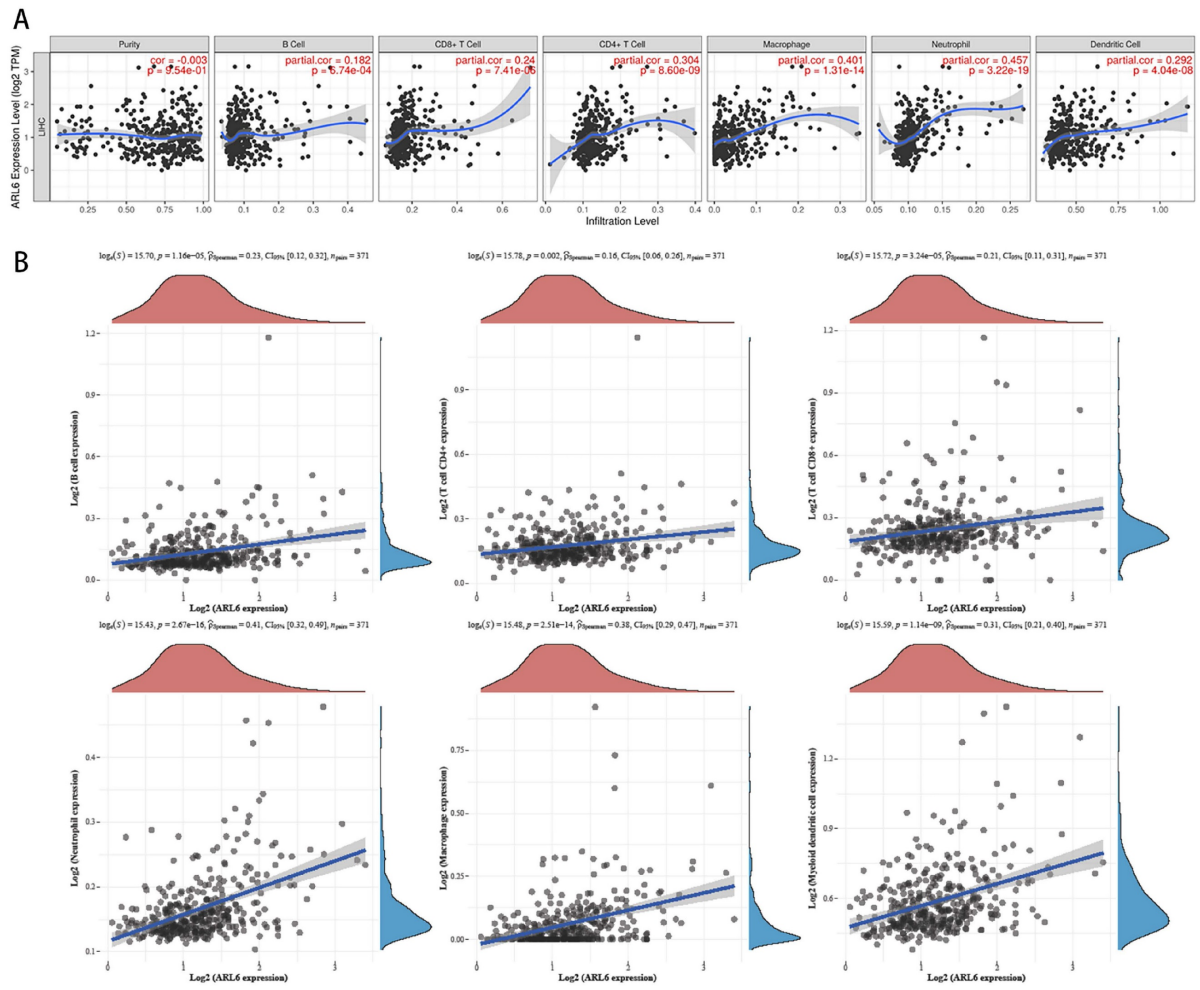
Immune infiltration levels and its correlation with ALR-6 expression in HCC. (A) The relationship between tumor immune microenvironment (Dendritic cells, neutrophils, macrophages, and CD4+T, CD8+T and B cells) and *ARL-6* in TIMER database. (B) The TCGA database was analyzed for *ARL-6* expression to determine the invasion of immune-associated cells.

We utilized the TIMER database to investigate the correlation between immune cell infiltration and variations in *ARL6* gene expression, as invading immune cells and the inflammatory response could affect HCC prognosis. A significant association was found among *ALR6* expression and the infiltration of dendritic cells (Cor=0.292, P=1.95e-8), neutrophils (Cor=0.457, P=3.22e-19), macrophages (Cor=0.401, P=1.31e-14), CD4+ T cells (Cor=0.304, P=8.60e-9), CD8+ T cells (Cor=0.24, P=7.41e-6), and B cells (Cor=0.182, P=6.74e-4) (Figure 6A).

To further investigate the relationship with the human immune system, we examined the correlation between *ARL-6* expression and the HCC immune microenvironment according to the TCGA database. B cells (P=1.16e-5), CD8+T cells (P=3.24e-5), CD4+T cells (P=0.002), neutrophils (P=2.67e-16), macrophages (P=2.51e-14), and myeloid dendritic cells (P=1.14e-9) were significantly correlated with *ARL-6* expression in HCC (Figure 6B).

### Co-Expression and Functional Enrichment Evaluation of *ARL6* Gene in HCC Patients

**Figure 7:**
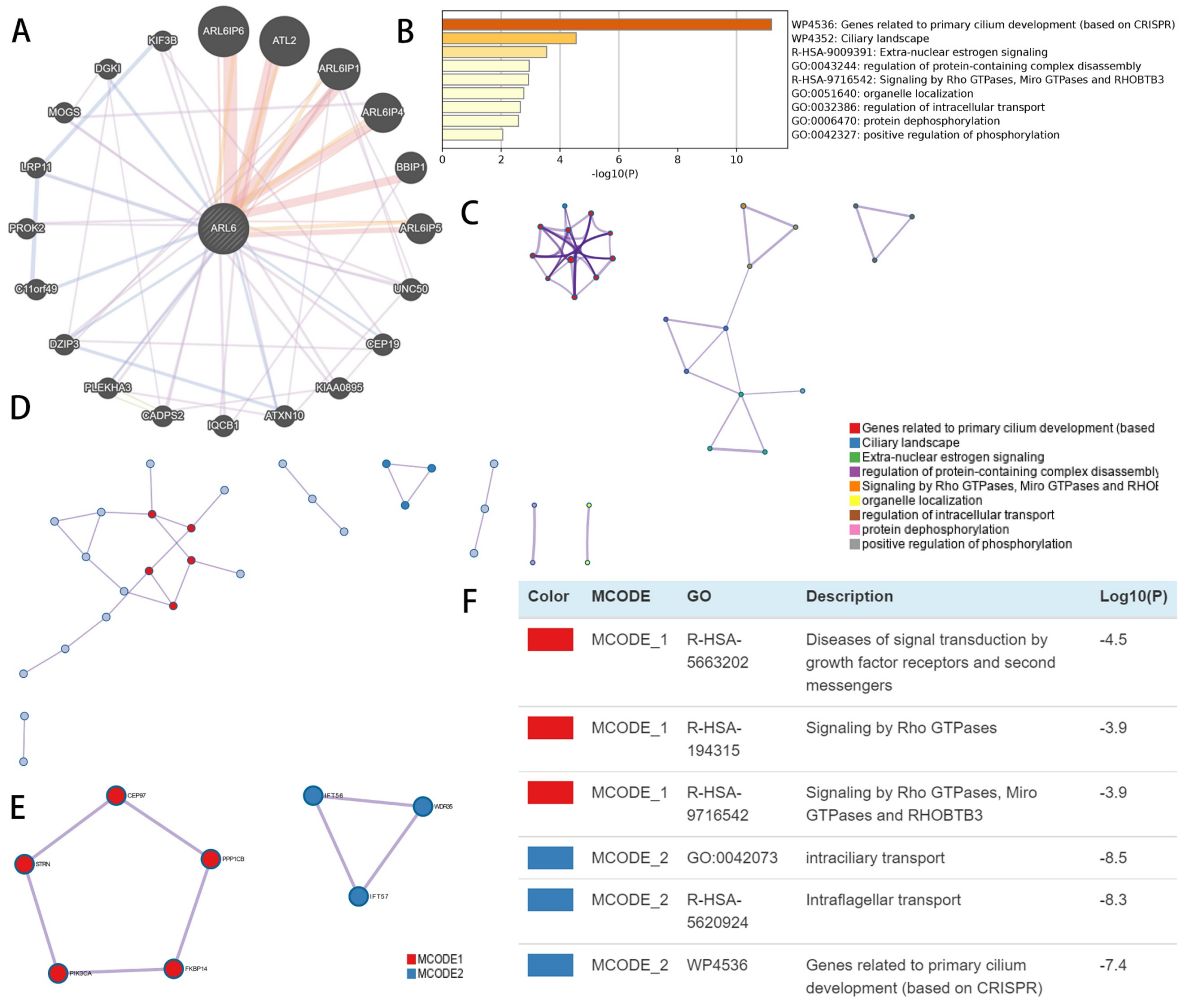
Functional enrichment and co-expression evaluation of *ARL-6* gene in HCC cases. (A) Gene-gene network of *ARL-6* gene was constructed from GeneMANIA database. (Graph (B) showing the top 9 enriched keywords for *ARL-6* and the top 50 co-expressed genes via the Metascape database. (C) Cluster IDs are utilized for coloring the enhanced phrases in the network. (D-F) Identifying the PPI network and the MCODE components.

Using GeneMINIA, we constructed an interconnected matrix of *ARL-6* and functionally associated genes to investigate the underlying mechanistic controls of *ARL-6* members in HCC. The analysis revealed that 20 genes, such as *ARL6IP6, ATL2, ARL6IP1, ARL6IP4, BBIP1, ARL6IP5, UNC50, CEP19, KIAA0895, ATXN10, IQCB1, CADPS2, PLEKHA3, DZIP3, C11*or*f49, PROK2, LRP11, MOGS, DGK1* and* KIF3B* (Figure 7A), were primarily associated with the modulatory functions of *ARL6* gene among patients with HCC.

Metascape was employed to analyze co-expressed genes with differentially expressed *ARL6* and determine their biological significance. The top nine enriched terms included genes related to primary cilium development, ciliary landscape, extra-nuclear estrogen signaling, regulation of protein-containing complex disassembly, signaling via *RHOBTB3*, Miro GTPases and Rho GTPases, organelle localization, regulation of intracellular transport, protein dephosphorylation and positive regulation of phosphorylation (Figure 7B). In addition, we constructed a network colored by ID with enhanced keywords. The association between *ARL6* differential expression and HCC was further explored using mCODE analysis and a protein-protein interaction network. This network was used to isolate essential mCODE components, revealing diseases associated with signal transduction via growth factor receptors and second messengers, as well as diseases associated with signaling via Rho GTPases, Miro GTPases, RHOBTB3, intraciliary transport, intraflagellar transport, and genes involved in primary cilium development (Figures 7C-F).

### Function of *ARL-6* on Proliferation, Apoptosis and Invasion of Hepatocellular Carcinoma Cells

**Figure 8:**
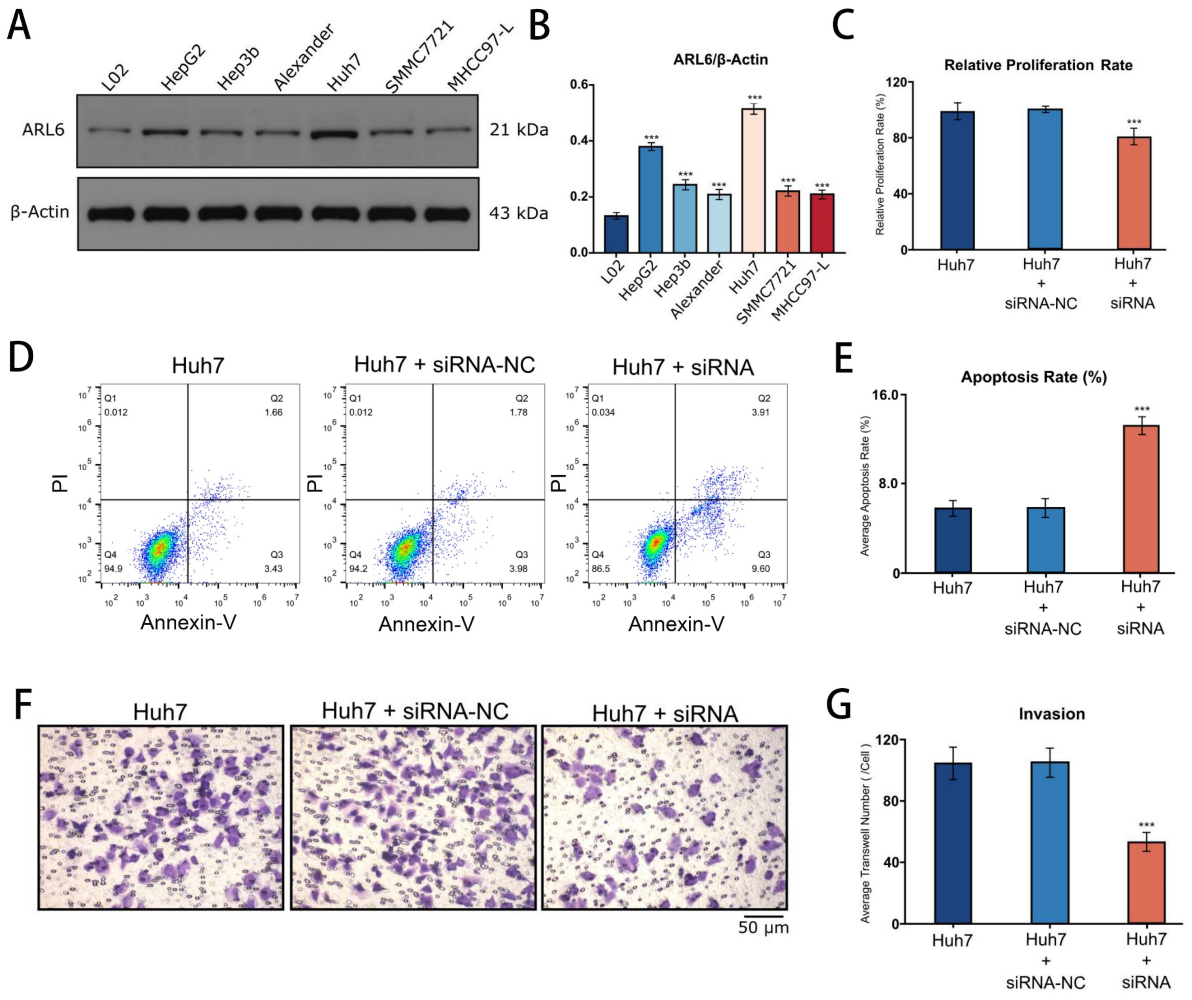
Cell proliferation, apoptosis, and invasion after the suppression of *ARL-6* gene by siRNA. (A-B) The expression of *ARL-6* in human normal and HCC cell lines. (C) CCK8 assay was employed for detecting cellular proliferation. (D-E) Scanning, quantifying, and plotting the flow cytometry assay pictures in D. After performing an invasion experiment, the pictures in (F) were scanned, counted, and plotted in (G). Significance: *p < 0.05; **p < 0.01; ***p < 0.001.

Western blotting revealed the expression of *ARL-6* in human liver cancer cell lines (Hep-G2, Hep3B, Alexander, Huh7, SMMC7721, and MHCC97-L), as well as in a human normal liver cell line (LO2). The highest expression of *ARL-6* was observed in the Huh7 cell line, although elevated levels were present in all of the HCC cell lines (Figure 8A). Subsequently, Huh7 cells were treated with siNC and *ARL6*-siRNA. Figure 8B shows that compared to siNC-treated and untreated cells, *ARL-6*-siRNA treatment led to a significant reduction in cell growth. Furthermore, cell apoptosis was significantly increased in LO2 cells treated with *ARL6*-siRNA compared to both siNC-treated and untreated cells (P<0.05; Figure 8C). *ARL6*-siRNA treated Huh7 cells demonstrated an obvious impact of* ARL6* on tumor invasion compared to the negative control groups (P<0.001, Figure 8D). Altogether, our results suggest the intriguing possibility that *ARL-6* plays a role in hepatocarcinogenesis by promoting the proliferation and spread of HCC cells. These findings provide further support for the idea that *ARL-6* contributes to the development and prognosis of HCC.

## Discussion

HCC typically progresses slowly, and its symptoms often become evident only in the middle or late stages of the disease. HCC is associated with a poor prognosis due to its high malignancy and resistance to radiation and chemotherapy 8, 16. Therefore, early and accurate detection and assessment of HCC lesions are critically important to improve prognosis and survival. *ARL-6* plays several potential roles in the therapeutic context of HCC. It serves as a promising prognostic biomarker, facilitating the identification of high-risk patients who may require more aggressive treatments or closer monitoring. The involvement of *ARL-6* in HCC development and its influence on immune cell infiltration make it a promising candidate for targeted cancer therapies. Specifically, *ARL-6* levels can be useful for patient stratification and monitoring treatment response. Our findings revealed that *ARL-6* had a significantly high expression in HCC. We also observed that increased *ARL-6* expression was associated with various tumor types, TP53 mutation status, nodal metastasis status, different cancer stages, and histological subtypes. Furthermore, in HCC patients, overexpression of *ARL-6* was linked to shorter OS and DFS. Additionally, *ARL-6* expression was shown to be a reliable predictor of OS in the 1^st^, 3^rd^, and 5^th^ years in the TCGA-HCC group, suggesting that *ARL-6* gene may serve as potential prognostic biomarkers in HCC. It is worth noting that our study analyzed data from multiple databases, and although we could not establish a definitive causal link between the extracted data, the expression of *ARL-6* in HCC was further validated through our single-center clinical samples. Our results showed that the *ARL-6* expression patterns in HCC were consistent with those analyzed from the database. Therefore, the overexpression of *ARL-6* gene might be utilized as a prognostic biomarker for HCC.

Rapid disease progression in HCC patients has been associated with insufficient immune cell infiltration into the tumor microenvironment 17, 18. Moreover, increased B cell infiltration has been linked to better outcomes in HCC, and recent research has confirmed that these tumor-infiltrating B cells may have a tumor-suppressive role in the HCC microenvironment 19. Xue et al. found that tumor-associated neutrophil (TAN) populations were associated with an unfavorable prognosis of HCC 20. Notably, our findings revealed a substantial association between *ARL-6* expression and cellular immune infiltration, shedding light on its potential significance in understanding HCC immunity. Exploring the TIMER database further revealed that *ARL-6* expression in HCC was significantly correlated with the infiltration levels of neutrophils, CD4+T, CD8+T and B cells, macrophages, and dendritic cells. Spearman's analysis of this relationship suggested a strong relationship between *ARL-6* expression and immune cell infiltration in HCC. However, there is still a knowledge gap regarding the precise function of *ARL-6* in the tumor immune microenvironment that requires further investigation.

Furthermore, we delved into the molecular mechanism of the *ARL-6* gene in HCC patients. Previous research has linked *ARL-6*'s mechanism of action in various diseases to its regulation of multiple signaling pathways 21-23. In this study, we identified key genes that may be associated with *ARL-6* function, and some of these genes were found to be significant regulators in HCC. For instance, increased KIF3B expression has been linked to worse OS, and it was observed that KIF3B expression was elevated in HCC tissues and proliferating cells 24. Another study implicated UNC50 in HCC development through its influence on the EGFR pathway 25, 26. Our analysis showed a significant correlation between *ARL-6* gene and *KIF3B* or *UNC50* gene. However, the exact role of *ARL-6* in HCC carcinogenesis, metastasis, cell proliferation, and apoptosis remains unclear. Through in vitro experiments, we found that *ARL-6* plays a vital role in cellular invasion, proliferation, and apoptosis, highlighting its significant regulatory function in the development and progression of HCC.

In conclusion, our results suggest that the *ARL-6* gene may play a role in controlling tumor growth and may have immunotherapeutic implications for HCC by impacting tumor prognosis and the cancer immune microenvironment. To fully understand the potential prognostic and therapeutic implications of the *ARL-6* gene in HCC, further research is needed to gain a deeper understanding of how it is regulated during tumor growth and development.

## Figures and Tables

**Table 1:** The mean density, H-score, and IRS of carcinoma and para carcinoma tissue specimens.

Index	Carcinoma tissues (N=26)	Paired para-carcinoma tissues (N=26)	t	P value
Mean Density (Mean±SD)	0.26±0.05	0.21±0.03	6.366	<0.0001
H-Score (Mean±SD)	159.86±27.99	131.36±21.77	4.759	<0.0001
IRS (Mean±SD)	6.46±1.98	4.92±1.72	3.953	<0.001
